# Supplementary figures and images for: LncRNA TUG1/miR-29c-3p/SIRT1 axis regulates endoplasmic reticulum stress-mediated renal epithelial cells injury in diabetic nephropathy model in vitro
Source: PLoS One. 2021 Jun 7;16(6):e0252761. doi: 10.1371/journal.pone.0252761 (PMC8183992; doi:10.1371/journal.pone.0252761)

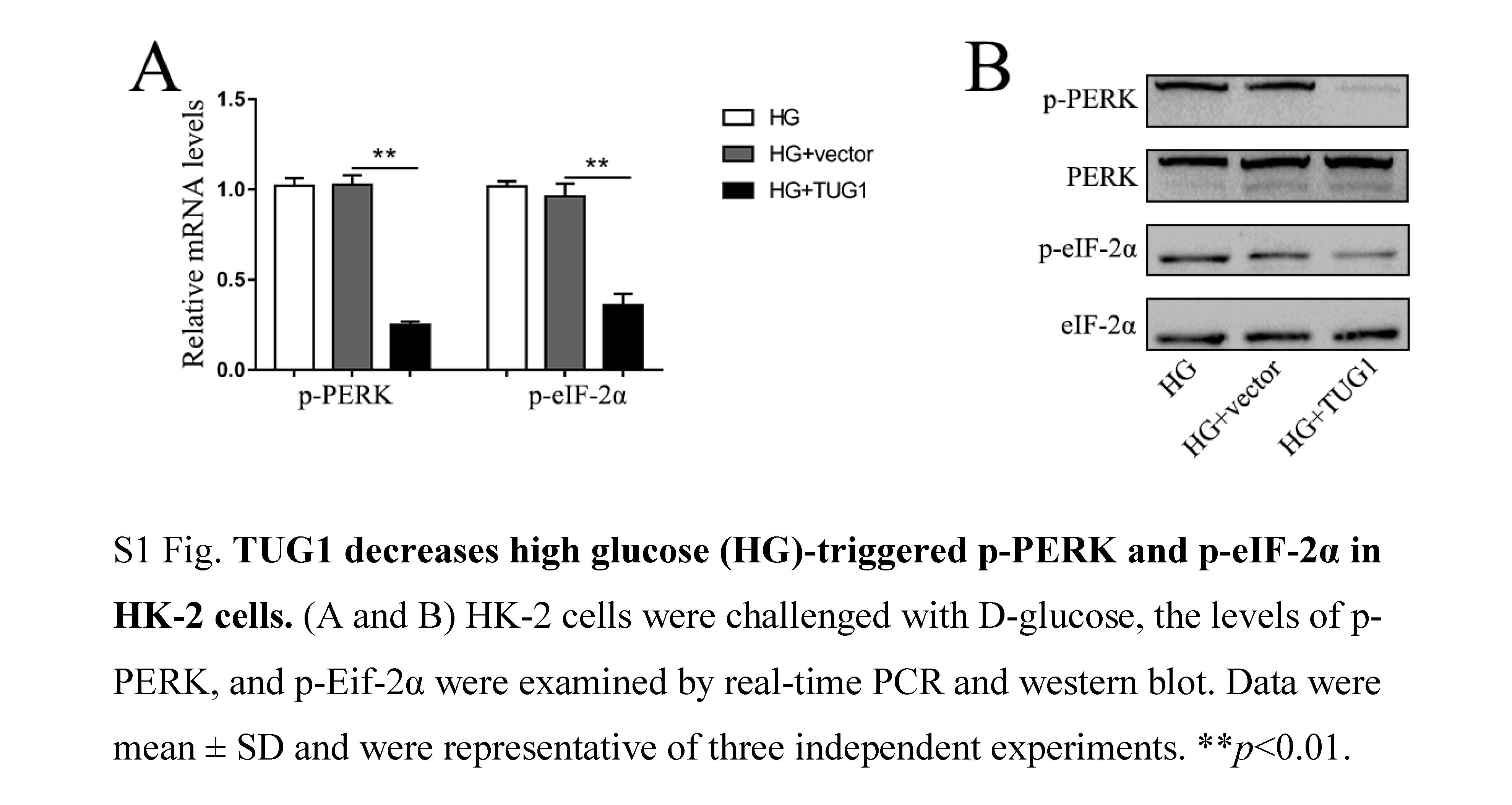

Supplement: S1 Fig — (A and B) HK-2 cells were challenged with D-glucose, the levels of p-PERK, and p-Eif-2α were examined by real-time PCR and western blot. Data were mean ± SD and were representative of three independent experiments. **p<0.01. (TIF) [file pone.0252761.s001.tif]

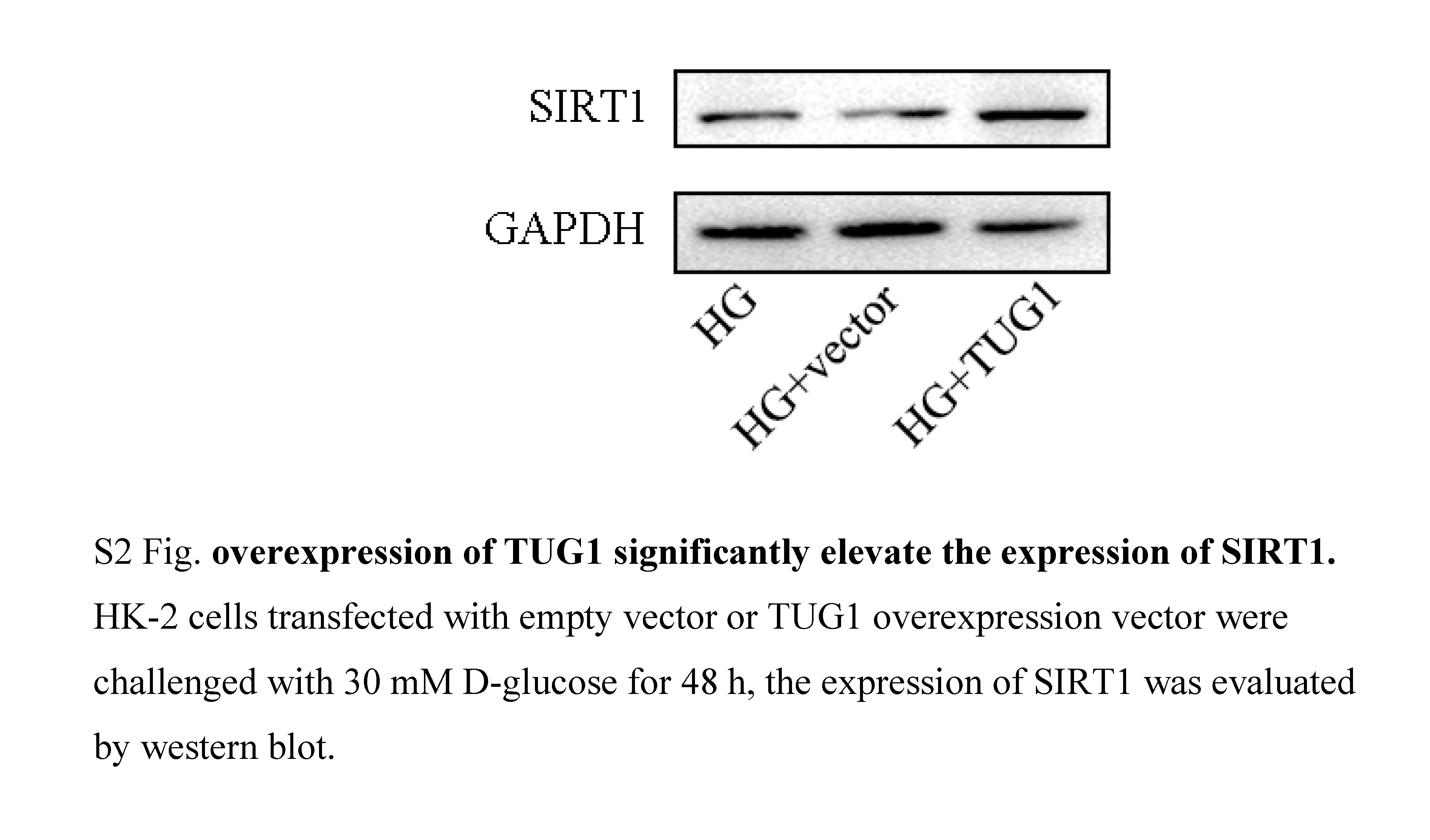

Supplement: S2 Fig — HK-2 cells transfected with empty vector or TUG1 overexpression vector were challenged with 30 mM D-glucose for 48 h, the expression of SIRT1 was evaluated by western blot. (TIF) [file pone.0252761.s002.tif]

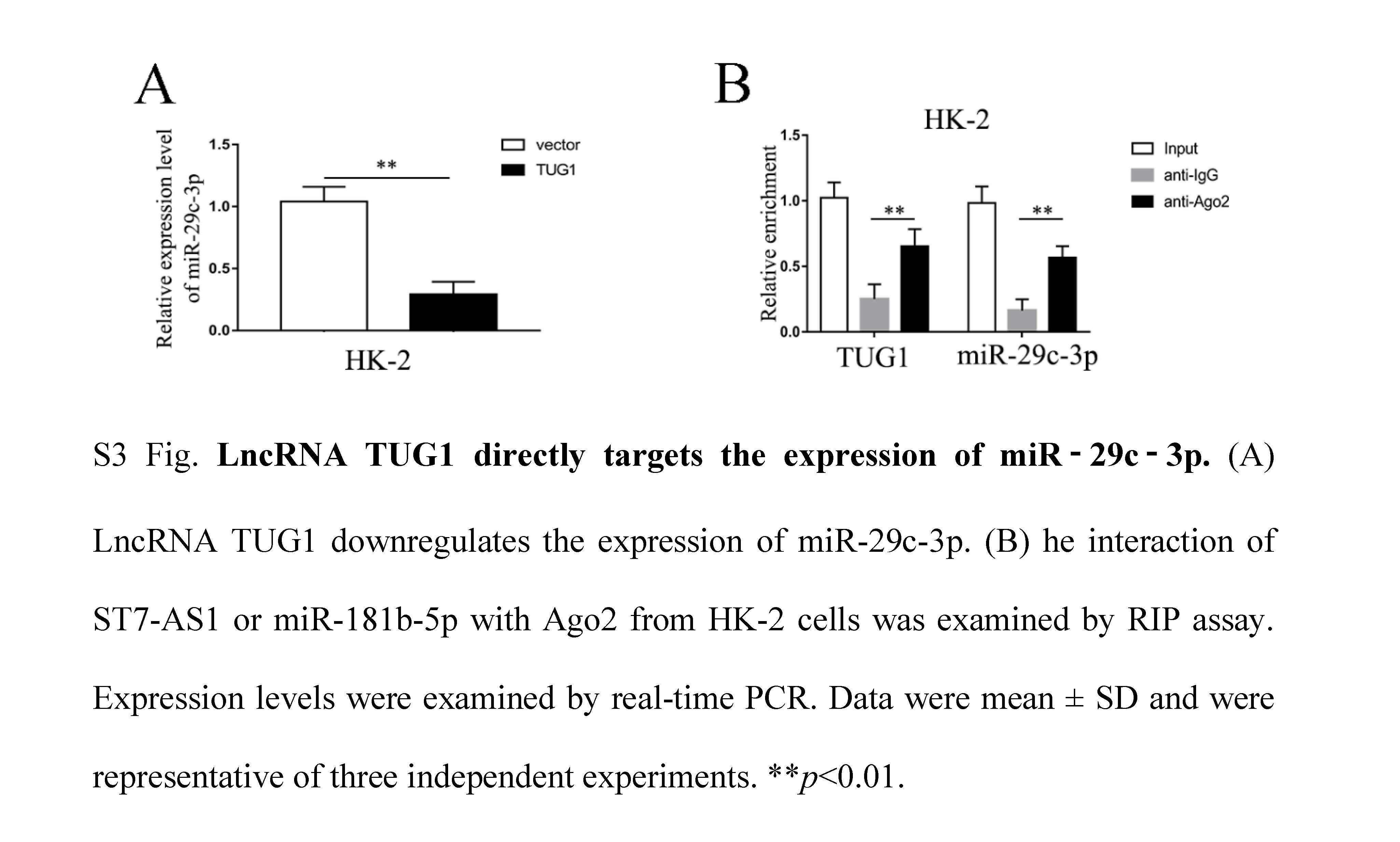

Supplement: S3 Fig — (A) LncRNA TUG1 downregulates the expression of miR-29c-3p. (B) he interaction of ST7-AS1 or miR-181b-5p with Ago2 from HK-2 cells was examined by RIP assay. Expression levels were examined by real-time PCR. Data were mean ± SD and were representative of three independent experiments. **p<0.01. (TIF) [file pone.0252761.s003.tif]

Fig 1

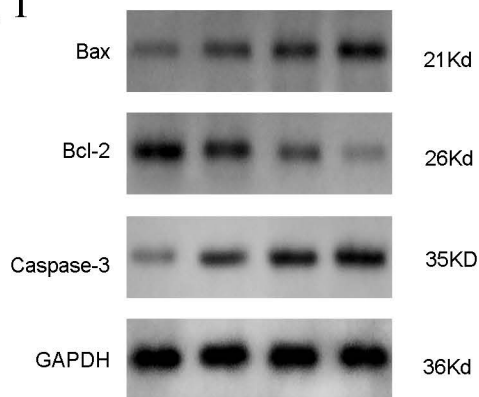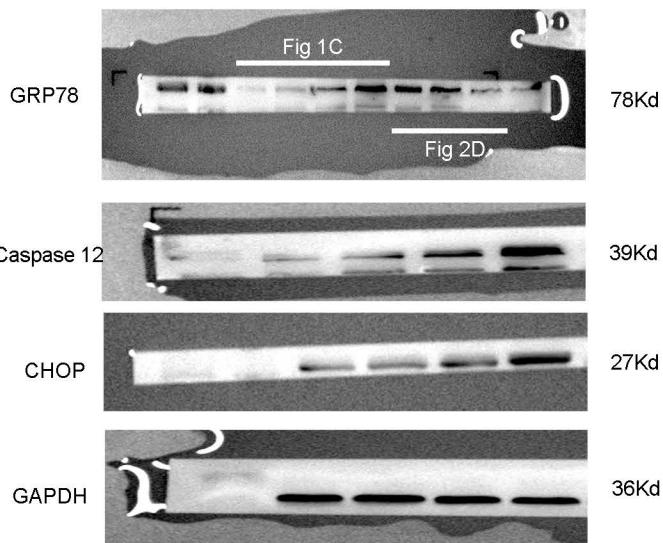

Fig 2

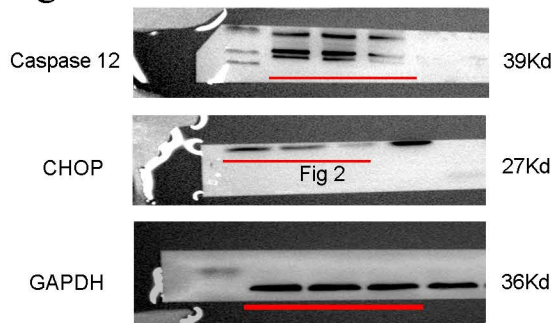

Fig 4A

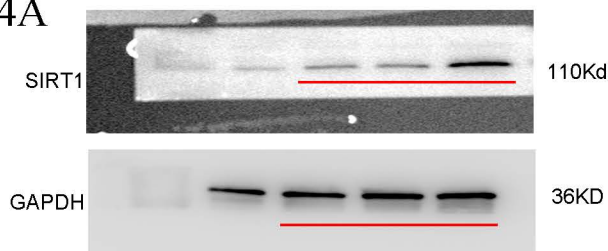

Fig 4C

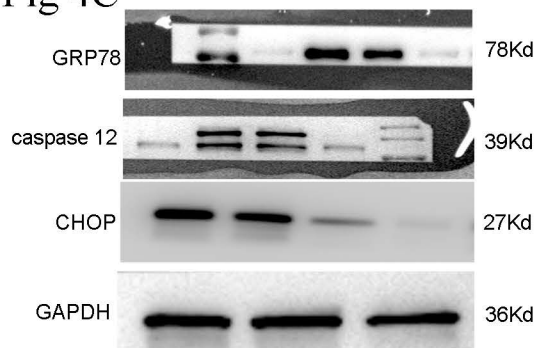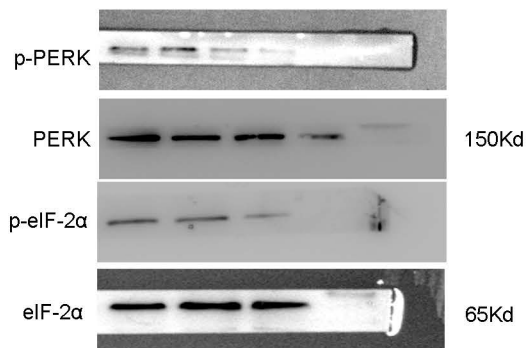

Fig 6A

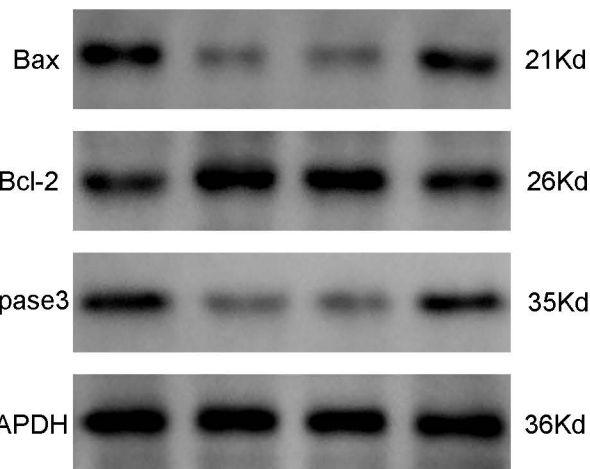

Fig 6B

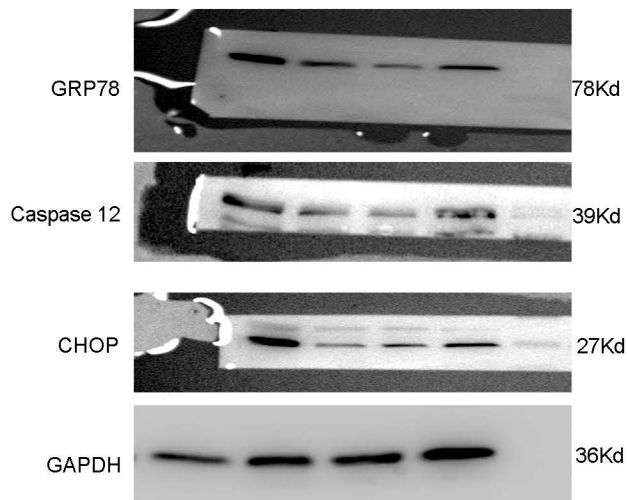

Fig 6C

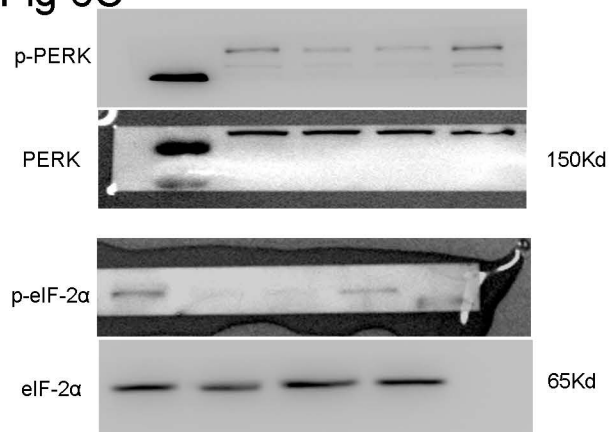

S1B Fig

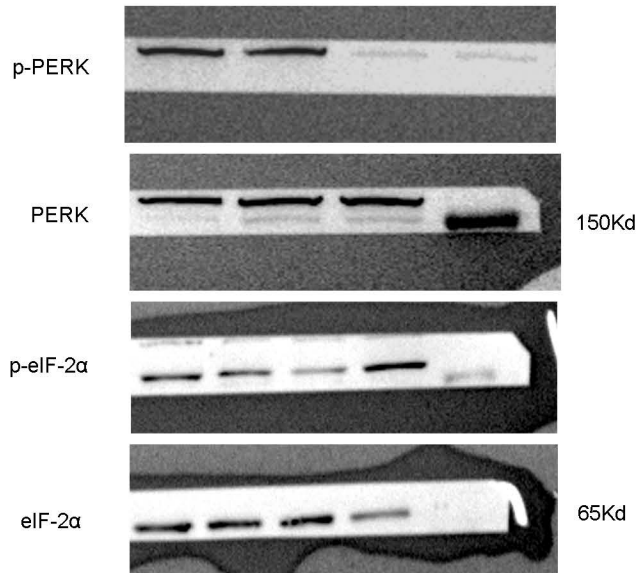

S2 Fig

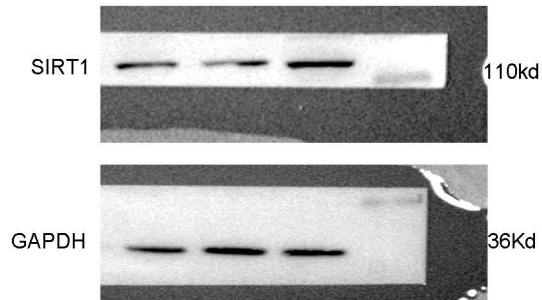

Supplement: S1 Raw images — (PDF) [file pone.0252761.s004.pdf]
