# Supplementary material for: LncRNA TUG1/miR-29c-3p/SIRT1 axis regulates endoplasmic reticulum stress-mediated renal epithelial cells injury in diabetic nephropathy model in vitro
Source: PLoS One. 2021 Jun 7;16(6):e0252761. doi: 10.1371/journal.pone.0252761 (PMC8183992; doi:10.1371/journal.pone.0252761)
Supplement: S1 File — (DOCX) [file pone.0252761.s005.docx]

Supplementary Figures

**LncRNA TUG1/miR-29c-3p/SIRT1 axis regulates endoplasmic reticulum stress-mediated renal epithelial cells injury in diabetic nephropathy model *in vitro***

Shaoqiang Wang^1^, Pengfei Yi^2^, Na Wang^2^, Min Song^2^, Wenhui Li^1^, Yingying Zheng^2*^

^
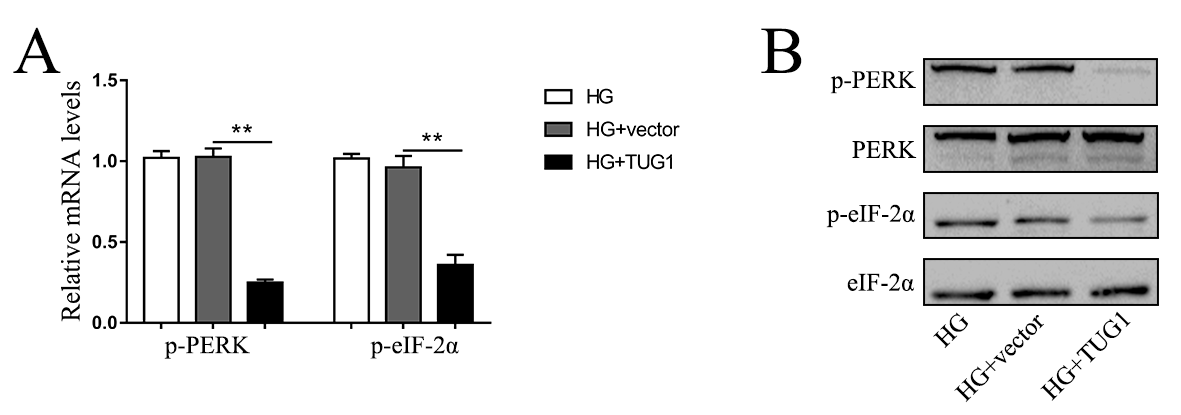
^

S1 Fig. **TUG1 decreases high glucose (HG)-triggered p-PERK and p-eIF-2****α in HK-2 cells.** (A and B) HK-2 cells were challenged with D-glucose, the levels of p-PERK, and p-Eif-2α were examined by real-time PCR and western blot. Data were mean ± SD and were representative of three independent experiments. ***p*<0.01.


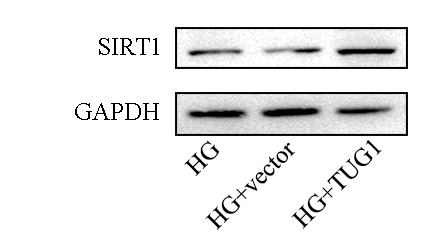


S2 Fig. overexpression of TUG1 significantly elevate the expression of SIRT1. HK-2 cells transfected with empty vector or TUG1 overexpression vector were challenged with 30 mM D-glucose for 48 h, the expression of SIRT1 was evaluated by western blot.


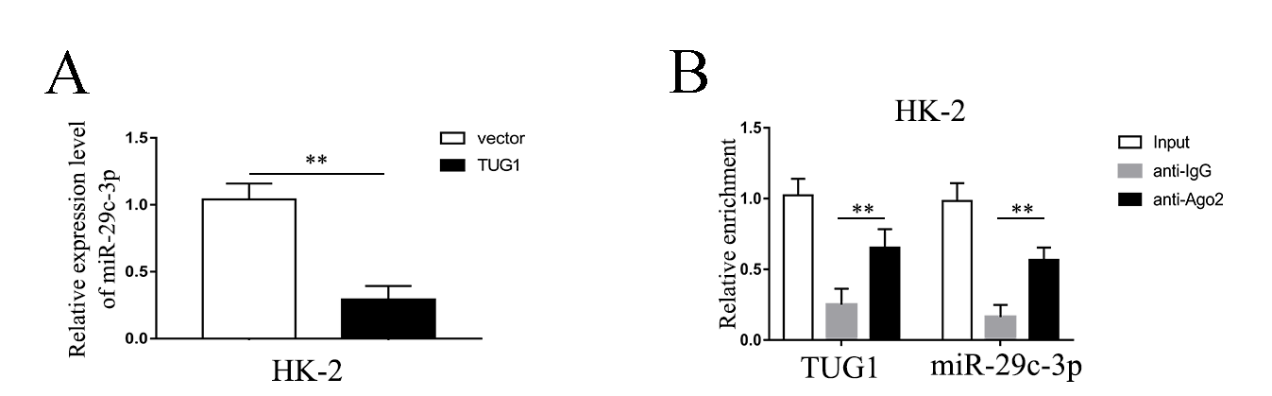


S3 Fig. LncRNA TUG1 directly targets the expression of miR‑29c‑3p. (A) LncRNA TUG1 down­regulates the expression of miR‑29c‑3p. (B) he interaction of ST7-AS1 or miR-181b-5p with Ago2 from HK-2 cells was examined by RIP assay. Expression levels were examined by real-time PCR. Data were mean ± SD and were representative of three independent experiments. ***p*<0.01.
